# Overestimation of physical activity level is associated with lower BMI: a cross-sectional analysis

**DOI:** 10.1186/1479-5868-7-68

**Published:** 2010-09-20

**Authors:** Clare Watkinson, Esther MF van Sluijs, Stephen Sutton, Wendy Hardeman, Kirsten Corder, Simon J Griffin

**Affiliations:** 1 MRC Epidemiology Unit, Institute of Metabolic Science, Addenbrookes Hospital, Hills Road, Cambridge, UK; 2 Department of Public Health and Primary Care, University of Cambridge, Cambridge, UK

## Abstract

**Background:**

Poor recognition of physical inactivity may be an important barrier to healthy behaviour change, but little is known about this phenomenon. We aimed to characterize a high-risk population according to the discrepancies between objective and self-rated physical activity (PA), defined as awareness.

**Methods:**

An exploratory cross-sectional analysis of PA awareness using baseline data collected from 365 ProActive participants between 2001 and 2003 in East Anglia, England. Self-rated PA was defined as 'active' or 'inactive' (assessed via questionnaire). Objective PA was defined according to achievement of guideline activity levels (≥30 minutes or <30 minutes spent at least moderate intensity PA, assessed by heart rate monitoring). Four awareness groups were created: 'Realistic Actives', 'Realistic Inactives', 'Overestimators' and 'Underestimators'. Logistic regression was used to assess associations between awareness group and 17 personal, social and biological correlates.

**Results:**

63.3% of participants (N = 231) were inactive according to objective measurement. Of these, 45.9% rated themselves as active ('Overestimators'). In a multiple logistic regression model adjusted for age and smoking, males (OR = 2.11, 95% CI = 1.12, 3.98), those with lower BMI (OR = 0.89, 95% CI = 0.84, 0.95), younger age at completion of full-time education (OR = 0.83, 95% CI = 0.74, 0.93) and higher general health perception (OR = 1.02 CI = 1.00, 1.04) were more likely to overestimate their PA.

**Conclusions:**

Overestimation of PA is associated with favourable indicators of relative slimness and general health. Feedback about PA levels could help reverse misperceptions.

## Background

While the public health importance of physical activity is well established[1,2], levels of physical activity in the UK have continued to decline and only a third of the population currently meet minimum recommendations[3]. A growing body of research has been directed towards physical activity interventions, but recent reviews show limited evidence of sustained behaviour change and the underlying barriers remain unclear[4-6].

One possible barrier is that sedentary individuals may be unaware of their inactivity. Unlike dichotomous behaviours such as smoking, physical activity spans multiple planned, incidental and habitual activities over a 24-hour period and thresholds of healthy versus unhealthy behaviour may be less clear[7]. This is particularly true of moderate activity (e.g. walking, stair climbing etc), which is often habitual or incidental and may be more difficult to estimate than strenuous activity. Realistic self-assessment depends on accurate recall of the intensity, frequency and duration of physical activity episodes, as well as knowledge of current guidelines and an appropriate definition of physical activity-all requiring high levels of physical activity salience. Evidence from dietary research suggests that summation of this complexity into a single global index may be subject to significant error [8-10], with misperceptions either facilitating (via underestimation) or hindering (via overestimation) behaviour change.

Thresholds of perceived inactivity may also have declined over recent decades, contributing to poor recognition of unhealthy behaviour. Little is known about this issue in relation to physical activity, but international weight perception data suggest that the increased prevalence of obesity over the last decade has been paralleled by a reduction in the ability to self-diagnose overweight [11-13]. Rising inactivity over recent decades may have reduced peoples' ability to distinguish low physical activity levels in a similar way, perhaps creating a faulty social perception that sedentary lifestyles are normal and sufficient. Indeed, work by Lechner et al suggests that Overestimators are more likely to rate their physical activity via comparison with others[14]. With less than 35% of the UK currently active [3], however, such strategies may be misleading.

Evidence to date indicates that more than 60% of adults who do not currently meet recommended guidelines overestimate their level of physical activity, and overestimation is more likely among those with a lower BMI [5]. Moreover, only 27% of overestimators reported a positive intention to change behaviour, compared to 43% among those who accurately assessed their inactivity [5]. Despite being at greatest risk, those who fail to recognise their inactivity are unlikely to perceive a need to change [9,10] and may therefore be less susceptible to health promotion strategies.

To date, however, misperceptions about physical activity in adults have been assessed by comparing two types of self-report measures; self-rated and quantified self-report [5,7,14]. A self-rated measure asks respondents to rate their PA behaviour by selecting one response from a simple scale of options i.e. a single overall summary score of their general PA behaviour. A self-reported measure summarises detailed quantified recollections of PA behaviour over a defined time period (e.g. past week/month/year), usually by means of questionnaires or diaries. Answers to both are used separately to score adherence to PA guidelines [15]. In the past, discrepancies between self-rated and self-reported guideline adherence have been used to determine 'awareness' of physical activity behaviour, and differences with objectively measured physical activity have only been considered in the context of questionnaire error and validity. In this study, the potential discrepancy between objectively measured and self-rated PA is the variable of interest. We are not looking to examine the validity of self-rated versus objective PA, but to examine participants' awareness of the adequacy of their overall PA behaviour (self-rated) compared with objective values. Due to potential error from shared method variance between self-rated and self-reported PA levels, the use of objective physical activity measurement for quantification of PA levels, rather than self-report, would give greater validity to awareness assessment.

Using baseline data from the ProActive cohort [16], we compare objective and self-rated measures of physical activity among sedentary individuals at high-risk of developing diabetes. We undertake an exploratory analysis to examine which personal, social and biological factors are associated with overestimation, and what role psychological variables might play. Results should help confirm whether or not previously reported associations persist when an objective measure of physical activity is used, and will help facilitate identification of Overestimators.

## Methods

### Study design and participants

In brief, ProActive aimed to evaluate the efficacy of a theory and evidence-based intervention programme to increase physical activity in a self-defined sedentary population. Those aged 30-50 years who had a parental history of diabetes but no known diabetes themselves were eligible to take part. 1521 potentially eligible people were identified via 20 general practices in East Anglia, England [16]. Of these, 1123 completed and returned a brief screening questionnaire based on published measures of occupational and leisure activity [17,18]; 286 were unwilling to participate and 343 were excluded because they were highly active (as defined by the questionnaire). A further 29 were excluded because either they had been prescribed β blockers that affected heart-rate variability; were unable to walk briskly across flat terrain for 15 min; lived further than 30 min by car from the study centre; or had illness or social obligations that would prevent participation. The remaining 465 potentially eligible participants were screened by telephone to check eligibility and confirm willingness to participate; 31 did not meet inclusion criteria and 35 refused to participate. Although baseline measurements were taken for 399 people, 24 of these participated in the pilot study and a further 10 were excluded prior to randomisation (seven did not meet inclusion criteria, two were unwilling to participate, and one agreed to participate after recruitment had closed). Full details of the original ProActive Trial intervention and protocol have been reported elsewhere [16].

All participants attended one of two measurement centers at baseline, where physiological and anthropometrical measures were taken and participants completed self-administered questionnaires. Immediately post-visit, all participants were measured using individually calibrated heart-rate (HR) monitoring over four consecutive days.

Ethical approval was obtained from the Eastern England MREC and all participants gave written informed consent.

### Measures

#### Awareness

Physical activity awareness was defined as the agreement between self-rated and objectively-measured activity according to current guidelines. Self-rated physical activity was assessed using the following question: 'In general, over the last year would you say you have been: extremely active/moderately active/not very active?' Respondents were classified as 'active' if they answered either 'extremely active' or 'moderately active', and inactive if they answered 'not very active'. Objective free-living physical activity was measured using individually calibrated HR monitoring. The method has been validated against the gold standard techniques of doubly labelled water and whole-body calorimetry [19] and is strongly associated with cardiovascular fitness [20] and the metabolic syndrome [21,22]. Participants wore HR monitors (Polar Electro, Kemple, Finland) continuously during the waking hours of the four consecutive days post-visit, and were classified as 'active' if they spent more than 30 minutes per day above 1.75 times resting HR (taken as the best approximation of moderate to vigorous physical activity [23]).

**Figure 1:**
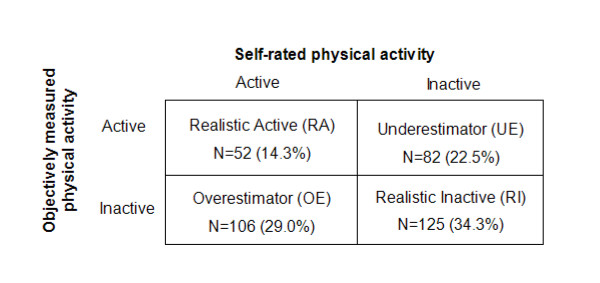
Classification of participants into awareness categories (N = 365)

In the absence of literature on HR monitoring duration, the choice of a four-day measurement period was based on evidence suggesting that between three and five days of monitoring is necessary to reliably assess habitual objective physical activity levels in adults when using accelerometry [24]. All information was concealed inside the monitor and volunteers did not have access to their HR data at any point. Access and interpretation of the data required specialized software for downloading and processing (available only to the research team). To assess a daily average of minutes of moderate and vigorous physical activity, the fraction of time spent above 1.75 times resting HR was multiplied by the number of minutes recorded and divided by four (the number of wear days). As data on hours worn per day were unavailable, we assumed an average of 12 hours per day for all participants and planned sensitivity analyses to test this assumption. Participants were defined as 'active' if they achieved an average of at least 30 minutes per day (≥ 4.17% of recorded time, calculated as 30 minutes divided by 720 minutes of daily wear time) spent above 1.75 times resting heart rate. Self-rated and objectively-measured physical activity levels were then grouped in a 2 × 2 table to create four awareness categories: 'Realistic Actives', 'Realistic Inactives', 'Overestimators' and 'Underestimators' (Figure 1). The analysis was also repeated with different thresholds (5% either side of the cut-off point) for classifying active versus inactive participants.

#### Potential correlates of awareness

Psychosocial correlates were measured using a self-administered questionnaire based on the Theory of Planned Behaviour (TPB)[25-27] for which items were selected on the basis of an elicitation study in a similar target group [28]. Perceived behavioural control was assessed using two items: 'it would be difficult for me to be more physically active in the next 12 months even if I wanted to' and 'I am confident that I could be more physically active in the next 12 months, if I wanted to'. Behavioural intention included 'I intend to be more physically active in the next 12 months', and 'it is likely that I will be more physically active in the next 12 months'. Attitude comprised instrumental attitude (measured with two items: 'being more physically active in the next 12 months would be good/harmful for me') and affective attitude (two items: 'for me, being more physically active in the next 12 months would be enjoyable/boring'). Subjective norm was measured using two items: 'most people who are important to me would want me to become more physically active in the next 12 months', and 'most people whose views I value would disapprove if I was more physically active in the next 12 months'. Items were measured on a Likert scale ranging from 1 (strongly disagree) to 5 (strongly agree), based on recommendations by Ajzen [25]. For each variable the scores for negatively formulated items were reversed and a mean score calculated across items for each participant.

Physiological correlates included weight, height and body fat percentage. Weight was measured on standard calibrated scales (SECA; London, UK) and height was determined using a rigid stadiometer. Body mass index (BMI) calculated as weight (kg) divided by height-squared (m^2^). Body fat percentage was measured by bio-electrical impedance (Bodystat, Isle of Man, UK). To provide an objective measurement of total physical activity energy expenditure, daytime physical activity ratio (DayPar - the ratio of daytime energy expenditure to resting energy expenditure) was also measured using heart rate monitoring with individual calibration for the heart rate-energy expenditure relationship [20].

Age at completion of full-time education, employment status (employed/unemployed), smoking habits (current/former/never) alcohol intake (units per week), and partner's leisure activity (same definitions as for self-rated physical activity) were all measured using a questionnaire developed for the study [16]. General health perception was measured as part of the SF-36 survey using a scale from 0 to 100, whereby higher scores denote more positive perceptions [29].

### Statistical analyses

An exploratory analysis was performed using Stata 8.0 software. All data were analysed as continuous variables where possible. To test for overall differences between the four awareness groups, Chi Square tests were applied for categorical variables (gender, employment and smoking groups), and analysis of variance was used for continuous data.

Analyses were undertaken separately for Active participants (Underestimators vs Realistic Actives) and inactive participants (Overestimators vs Realistic Inactives), with a predominant focus on the latter. Associations were modelled using forward-fitting step-wise logistic regression. Successive models were created by adding each independent variable according to ordered blocks; biological (age, sex, BMI, fitness), social/behavioural (education, employment, DayPar, smoking, alcohol intake, and partner's leisure activity over the last year, general health perception). Psychological variables were modelled separately to allow exploration of associations rather than causal pathways. Candidates for each model were identified by significant univariate associations (p ≤ 0.05).

## Results

### Sample characteristics

The mean age of participants was 40.6 years (SD 6.0) and mean BMI (kg/m2) was 27.8 (SD 5.1). Average daily time spent above 1.75 times resting heart rate showed a skewed distribution, with a mean of 5.64% and a median of 2.45% (SD = 8.28; Range = 0-48.6). Participants were generally overweight (68.2% had a BMI greater than 25), with 28% classified as obese (defined as a BMI of 30 or above), and reported health and anxiety levels comparable to population norms [29,30]. The majority were female (61.9%) and non-smokers (80.6%). 50.6% were in managerial or professional jobs, and mean age at completion of full-time education was 17.9 years (SD 3).

Of 365 participants, 63.3% were objectively classified as inactive (Figure 1). Almost half of these (45.9%) incorrectly rated themselves as active (Overestimators). In terms of the total sample, 29.0% were classified as Overestimators, 14.3% as Realistic Actives, 34.3% as Realistic Inactives (RI) and 22.5% as Underestimators. Repeating this analysis with a 5% change in threshold either side of the cut-off point did not affect the overall results (data not shown).

### Characteristics of awareness groups

Table 1 shows the results of the univariate analysis. Overestimators had a lower BMI and body fat percentage than Realistic Inactives (p < 0.005). Underestimators were the youngest group and had a higher BMI and body fat percentage than either Realistic Actives or Overestimators. A higher percentage of Realistic Actives (30.8%) and Underestimators (29.3%) were smokers, compared to only 9.6% of Realistic Inactives and 17.9% of Overestimators. General health perceptions were highest among Overestimators and lowest among Underestimators. Compared to those who were realistic about their inactivity, Overestimators scored higher on subjective norms.

**Table 1:** Cross-sectional associations between participant characteristics and physical activity awareness, by objectively measured activity (N = 365)

	ACTIVE (N = 134)			INACTIVE (N = 231)		
Participant characteristics	Realistic active (RA)	Under-estimator (UE)	P-value	Realistic inactive (RI)	Over-estimator (OE)	P-value
N (%)	52 (14.3)	82 (22.5)	-	125 (34.3)	106 (29.0)	-
						
***SOCIODEMOGRAPHIC***						
Gender (% male)^a^	42.3	48.8	0.464	28.8	38.7	0.113
Age in years (mean, SD)^b^	40.1 (5.5)	38.9 (6.0)	0.258	40.7 (6.1)	42.0 (5.9)	0.114
Age finished full time education (mean, SD)^b^	17.7 (2.6)	18.1 (3.3)	0.479	18.5 (3.7)	17.1 (2.5)	0.002
Percent unemployed (%) ^b^	2.0	2.5	0.833	2.4	3.8	0.553
Partner's leisure activity over last year ^a^			0.231			**0.012**
Some (%)	50	38.6		**34.7**	**53.6**	
Not much (%)	50	61.4		65.3	46.4	
						
***BIOLOGICAL***						
Body fat% (mean, SD)^b^	**28.6 (7.7)**	**32.3 (8.1)**	**0.013**	**33.4 (7.7)**	**29.1 (7.5)**	**< 0.001**
Body Mass Index in kg/m2 (mean, SD)^b^	**26.3 (4.5)**	**29.0 (4.9)**	**0.002**	**28.9 (5.5)**	**26.3 (4.4)**	**< 0.001**
DayPAR (mean, SD) ^b^	2.4 (0.7)	2.3 (0.6)	0.200	1.6 (0.4)	1.6 (0.4)	0.494
Predicted VO2max in L/min (mean, SD)^b^	3.4 (1.0)	3.4 (1.0)	0.693	3.1 (0.9)	3.0 (0.9)	0.873
						
***BEHAVIOURAL***						
Smoking (% smokers) ^a^	30.8%	29.3%	0.853	9.6%	17.9%	0.068
Total units of alcohol/week (mean, SD)^b^	8.4 (9.3)	8.8 (9.6)	0.781	6.0 (6.6)	6.3 (8.6)	0.702
Average percentage of time (per day) spent above 1.75 × resting HR (mean, SD)^b^	14.5 (10.7)	12.4 (9.0)	0.229	1.1 (1.2)	1.4 (1.2)	0.097
						
***PSYCHOLOGICAL***						
Affective attitude (mean, SD)^c^	4.0 (0.5)	3.9 (0.7)	0.284	3.9 (0.6)	3.8 (0.6)	0.543
Perceived behavioural control (mean, SD)^c^	3.8 (0.5)	4.0 (0.6)	0.203	3.9 (0.6)	3.7 (0.6)	0.159
Instrumental attitude (mean, SD)^c^	**4.5 (0.5)**	**4.6 (0.5)**	**0.008**	**4.3 (0.4)**	**4.4 (0.5)**	**0.047**
Subjective norm (mean, SD)^c^	**3.7 (0.6)**	**4.1 (0.6)**	**< 0.001**	**3.7 (0.6)**	**3.8 (0.5)**	**< 0.001**
Intention (mean, SD)^c^	3.8 (0.5)	3.9 (0.7)	0.296	3.7 (0.6)	3.6 (0.6)	0.067
SF-36 General health perception (mean, SD)^c^	**73.9 (14.5)**	**64.6 (19.0)**	**0.005**	**67.2 (18.7)**	**74.2 (16.3)**	**0.004**

Values are means and standard deviations (SD) for continuous variables and percentages within awareness groups for categorical variables. SD: standard deviation; BMI: Body Mass Index; V02 max: Maximal oxygen uptake; DayPAR: Daytime physical activity ratio; HR: Heart-rate.

All statistically significant differences between groups are reported

^a^: tested using Chi-square

^b^: tested using logistic regression

Table 2 shows the results of multiple logistic regression of awareness on personal, social and biological variables. Among inactive participants, those with a lower BMI, men and smokers were more likely to overestimate their physical activity. Overestimation was also associated with a higher general health perception, a lower age at completion of full-time education, and lower subjective norms. Among active participants (N = 134), underestimation was more likely among those with a higher BMI, lower general health perception and higher subjective norms.

**Table 2:** Multiple logistic regression of the association between participant characteristics and awareness, by objectively measured activity (N = 365)

	ACTIVE (N = 134) Underestimator vs Realistic Active*			INACTIVE (N = 231) Overestimator vs Realistic Inactive*		
	
	OR	95% CI	P-value	OR	95% CI	P-value
***Sociodemographic***						
Age finished full time education				0.83^c^	0.74-0.93	0.001
***Biological***						
Body Mass Index in kg/m2	1.14^a^	1.04-1.25	0.004	0.89 ^c^	0.84-0.95	0.001
Sex						
Female	-	-	-	1.0*	-	-
Male				2.11^c^	1.12-3.98	0.021
***Behavioural***						
Smoking						
Non-smokers	-	-	-	1.0*		
Smokers				2.71^c^	1.10-6.68	0.030
***Psychological***						
Subjective norm	3.79^b^	1.96-7.35	< 0.001	0.39^d^	0.21-0.71	0.002
SF-36 General health perception	0.97^a^	0.95-0.99	0.015	1.02^c^	1.00-1.04	0.026

* Reference category

^a^: Adjusted for sex, age, BMI & general health perception

^b^: Adjusted for sex and age

^c^: Adjusted for sex, age, BMI, age finished full-time education, general health perception and smoking

^d^: Adjusted for sex, age, instrumental attitude and intention

## Discussion

In this first study to assess adult awareness using an objective measure of physical activity, we observed that of the 63.3% of the ProActive cohort who were inactive at baseline almost half (45.9%) considered themselves to be active (Overestimators). Furthermore, gender, weight, general health perception and the opinions of significant others about one's physical activity level were all associated with the concordance between self-rated and objectively measured estimates. This may facilitate identification of Overestimators.

Compared to those who correctly rated themselves as inactive, Overestimators had lower BMI on average and tended to have a higher general perception of their health. They were also more likely to be male and less likely to report that significant others in their social environment would like them to become more active. In contrast, participants who underestimated their activity levels were more likely to have a higher BMI and to have a lower general perception of their health compared to those who correctly rated themselves as active, and those underestimating their physical activity level were more likely to report that others would like them to be more active. Given well-known links between weight and physical activity, one possible explanation for these observations is that Overestimators interpret their lower BMI as proof of adequate activity levels, such that overestimation is partly due to 'favourable' anthropometric indicators (whereas the reverse is true for Underestimators) [5,14]. It would have been preferable to include more predictors of body image and body composition to explore this association further. That this phenomenon persists even in a sedentary population at high risk of type 2 diabetes is particularly noteworthy. However, further work using objectively assessed awareness would be necessary to better to estimate the generalizability of these results. How people feel about their general health may also bias their perception of being active or not. Those who 'feel' healthy might conclude that they do enough activity, while those who rate their overall health more negatively may assume that they are not doing enough. Alternatively, believing one-self to be physically active may by itself prompt feelings of health and well-being, irrespective of objective reality. Longitudinal designs would help explore these associations in more depth.

Men were more likely to overestimate their physical activity than women, possibly reflecting prevailing gender stereotypes i.e. that men are stronger, fitter and thus more 'physical' overall. Overestimators also tended to have spent less time in full-time education, and were less likely to report that significant others in their social environment would like them to become more active. Targeting the 'significant others' of inactive people may therefore offer a novel strategy for promoting physical activity, particularly among men.

Our results replicate previously reported associations between overestimation of physical activity levels, favourable indicators of weight status and lower subjective norms, and suggest that these are unlikely to be due to chance [5,7,14].While the strength of previous evidence was limited by its reliance on self-report, the current study demonstrates that these associations persist even when objective behavioural outcomes are used as a benchmark.

Prevalence of overestimation among inactive individuals is slightly lower in the current study (46%) compared to previously published papers, where figures range from 48% to 61% [7,10]. This may be attributable to the reliance in previous work on self-reported physical activity as the criterion method for defining awareness (since self-reported physical activity tends to produce overestimates of true physical activity), or to the use of different cut-off points for classifying active vs inactive volunteers. Differences in sample characteristics may also have played a role; as part of their recruitment, participants in *ProActive *were told that they were inactive and at a higher risk of diabetes, but they could reduce their risk by behaviour change. In contrast, two out of three of the previous studies were population-based. It is possible, however, that the discrepancy derives simply from the different criterion measures used (objective vs self-report), and given the greater validity of objective HR monitoring over self-report methods of estimating physical activity, the current estimates may be more reliable.

The strength of this study is the objective measurement of physical activity. Although the 30 minute cut-off point is somewhat artificial, objective physical activity data is likely to reflect true physical activity levels more accurately than self-report measures. It also avoids the problem of correlated error that arises when using two self-report measures. Although we were limited by the absence of data on monitoring duration per day, sensitivity analyses showed that assuming either 10 or 14 hours monitoring per day did not alter the overall findings. It is important to acknowledge, however, that self-rated physical activity was assessed opportunistically using the best measure collected at the time, and that this did not specifically assess participant perception of adherence to the physical activity guidelines assessed with the objective measure. It is unknown which question may best assess physical activity awareness, and which frames of reference are most prevalent in the population. However, as the self-rated physical activity assessment used here has an 'open' frame of reference and leaves interpretation up to the respondent, we feel that this is an appropriate measure given the current evidence base. Although it is likely that perceptions of 'extremely', 'moderately' or 'not very' active are strongly influenced by awareness and knowledge of the guidelines, our findings are however limited by the assumption that the active/inactive distinction for the self-rated and objective measures correspond to each other.

Observed discrepancies between self-rated and objective measures could also reflect a difference in time reference periods (self-rated physical activity over the past year versus objective physical activity over four days), but as these would be equal across groups it is unlikely to have affected overall findings. Discrepancies might also reflect the days of the week or season of the year in which participants were monitored; monitors were attached at the end of the visit and worn over the following 4 days, such that volunteers measured on Mondays and Tuesdays only have weekday free-living data available. Such variations are unlikely to have affected the main findings however, since ProActive participants were recruited and tested throughout the year and clinical visit days were randomly assigned.

We cannot rule out self-selection bias in this study. Participants in a physical activity intervention trial are likely to have had more interest in physical activity than non-responders, and Overestimators and Realistic Actives might have been excluded via the screening questionnaire. While this may have constrained the distribution, the observed range of physical activity was still quite large (mean fraction of time spent above 1.75 times resting HR was 5.6, with values ranging from 0 to 48.6 and a standard deviation of 8.3). Although the number excluded (30%) corresponds well with the proportion designated as active in UK prevalence surveys [3], it is important to keep in mind that ProActive participants were age-restricted and defined as sedentary and at-risk of type 2 diabetes through a parental history of the disease; they were not therefore representative of the general population. As we would expect to see an elevated prevalence of inactivity in ProActive, the true prevalence of overestimation may be higher than our results suggest and more in line with previous findings [5,7,14].

Finally, our cross-sectional design precludes the establishment of causality, and prospective cohort studies are recommended for future research. Other unmeasured factors may also play a role. It is also important to recognise that although objective assessment of physical activity has advantages over questionnaire assessment, estimation of this complex behaviour remains a challenge. Reactivity may vary between individuals, for example, and HR monitoring can be susceptible to poor pick-up or interference during free-living conditions [31] and is responsive to triggers such as stress, heat and caffeine. It must also be remembered that a person's heart rate is a physiological response to stimuli, whereas physical activity is a behaviour; although HR monitoring provides a useful guide for estimating physical activity, it is still a proxy measure rather than a direct one.

### Public health and research implications

Since public health messages are unlikely to reach those who do not recognise themselves as targets, our findings suggest that up to half of the inactive population may be inaccessible to physical activity interventions. Longitudinal research will help to establish the causal pathways involved, but raising physical activity awareness and adapting public health messages may be an important first step. Future messages could emphasize the benefits of physical activity beyond weight control and stress that inactive people may also be slim, although they are less likely to be healthy. Reversing misperceptions at the population level (i.e. collective versus high-risk interventions) may be the most effective way of reaching Overestimators.

Despite the cross-sectional nature of our findings, it is possible that measurement, awareness and feedback could offer new and interesting avenues for future intervention research. It has been shown that increased self-awareness may improve self-regulation of behaviour[32], and our study aimed to gain more insight into factors which might help us identify ways to help people become more aware of their behaviour. Further work is necessary to assess a) the relationship between awareness and behaviour, and between change in awareness and change in behaviour, and b) the effect of feedback on awareness and behaviour. Measurement and feedback have been associated with more accurate perceptions of behaviour and stronger intentions to change [33,34], and there are positive indications of behavioural effects. A number of randomised controlled trials reported unexpected physical activity increases in the control group indicative of a measurement effect [35-38], and there is promising evidence that pedometers can facilitate healthy behaviour change [39-41]. As such, measurement and feedback strategies could help Overestimators to recognise their own unhealthy behaviour and recalibrate their perceptions of inactivity. To explore this possibility, future research should aim to measure the effect of measurement and/or feedback on awareness and behaviour change, ideally via a randomised controlled trial.

## Conclusions

Almost half of inactive participants in this study incorrectly perceived themselves as 'active'. Overestimation of physical activity, defined here as the discordance between objective and self-rated physical activity, was associated with male sex, lower BMI, younger age at completion of full-time education and higher general health perceptions. These results replicate previously reported associations between physical activity overestimation and favourable indicators of health, and highlight the need for further longitudinal research. Strategies for facilitating realistic self-definition of physical activity level also warrant investigation.

## Competing interests

The authors declare that they have no competing interests.

## Authors' contributions

SJG, EMFvS and CW conceived and led the research. All authors contributed to the conception, design and interpretation of data. CW conducted data analyses and wrote the initial manuscript. All authors contributed to the critical revision of draft manuscripts and read and approved the final manuscript.
